# Sonothrombolysis with BR38 Microbubbles Improves Microvascular Patency in a Rat Model of Stroke

**DOI:** 10.1371/journal.pone.0152898

**Published:** 2016-04-14

**Authors:** Nadine Schleicher, Amelia J. Tomkins, Marian Kampschulte, Jean-Marc Hyvelin, Catherine Botteron, Martin Juenemann, Mesut Yeniguen, Gabriele A. Krombach, Manfred Kaps, Neil J. Spratt, Tibo Gerriets, Max Nedelmann

**Affiliations:** 1 Heart and Brain Research Group, Justus-Liebig-University, Giessen, Germany; 2 Department of Neurology, Justus-Liebig-University, Giessen, Germany; 3 Department of Cardiac Surgery, Kerckhoff Clinic, Bad Nauheim, Germany; 4 School of Biomedical Sciences & Pharmacy, University of Newcastle, and Hunter Medical Research Institute, Newcastle, NSW, Australia; 5 Department of Diagnostic and Interventional Radiology, Justus-Liebig-University, Giessen, Germany; 6 Bracco Suisse S.A., Plan-les-Ouates, Geneva, Switzerland; 7 Hunter New England Local Health District, New Lambton, NSW, Australia; 8 Department of Neurology, Buergerhospital Friedberg, Friedberg, Germany; 9 Sana Regio Klinkum, Pinneberg, Germany; 10 Department of Neurology, University Hospital Center Hamburg-Eppendorf, Hamburg, Germany; Stanford University, UNITED STATES

## Abstract

**Background:**

Early recanalization of large cerebral vessels in ischemic stroke is associated with improved clinical outcome, however persisting hypoperfusion leads to poor clinical recovery despite large vessel recanalization. Limited experimental sonothrombolysis studies have shown that addition of microbubbles during treatment can improve microvascular patency. We aimed to determine the effect of two different microbubble formulations on microvascular patency in a rat stroke model.

**Methods:**

We tested BR38 and SonoVue® microbubble-enhanced sonothrombolysis in Wistar rats submitted to 90-minute filament occlusion of the middle cerebral artery. Rats were randomized to treatment (n = 6/group): control, rt-PA, or rt-PA+3-MHz ultrasound insonation with BR38 or SonoVue® at full or 1/3 dose. Treatment duration was 60 minutes, beginning after withdrawal of the filament, and sacrifice was immediately after treatment. Vascular volumes were evaluated with microcomputed tomography.

**Results:**

Total vascular volume of the ipsilateral hemisphere was reduced in control and rt-PA groups (p<0.05), but was not significantly different from the contralateral hemisphere in all microbubble-treated groups (p>0.1).

**Conclusions:**

Microbubble-enhanced sonothrombolysis improves microvascular patency. This effect is not dose- or microbubble formulation-dependent suggesting a class effect of microbubbles promoting microvascular reopening. This study demonstrates that microbubble-enhanced sonothrombolysis may be a therapeutic strategy for patients with persistent hypoperfusion of the ischemic territory.

## Introduction

Transcranial ultrasound combined with microbubbles augments thrombolysis in clinical studies and experimental animal models of acute intracranial arterial occlusion [1–4]. Application of transcranial color-coded duplex sonography (TCCS) in conjunction with IV recombinant tissue-type plasminogen activator (rt-PA) and microbubbles reduces time to recanalization and increases the total numbers of patients with recanalization [2, 3]. In addition to large vessel recanalization, this therapy has the potential to increase perfusion of the cerebral microvasculature in areas of hypoperfusion [4].

Microbubbles are ultrasound contrast agents used diagnostically and, when administered with rt-PA and ultrasound, have the potential to improve thrombi dissolution [5]. Although the mechanism of thrombus dissolution by ultrasound and microbubbles is not fully understood, it has been previously hypothesised that administration of microbubbles dramatically lowers the threshold for ultrasound-induced cavitation that causes mechanical stress on thrombi, leading to destabilisation and subsequent thrombus dissolution [5, 6]. Hence, microbubbles lower the energy requirements for producing sonothrombolysis thereby increasing the lytic activity of ultrasound [6].

Despite successful recanalization of large arteries, the affected ischemic territory may remain hypoperfused. This is likely due to a “no reflow phenomenon” of the microcirculation whereby fibrin deposits and cell detritus occlude small cerebral vessels during the stagnant flow conditions of ischemia [7, 8]. While large vessel recanalization is an important therapeutic approach for stroke, growing evidence suggests that reperfusion is a better predictor of good clinical outcome [9–11]. The diagnostic ultrasound contrast agent, SonoVue®, containing sulfur hexafluoride microbubbles, has been shown to safely re-establish microcirculatory patency completely in an experimental study of stroke when administered in conjunction with TCCS and rt-PA [4]. Treatment with rt-PA alone did not restore microvascular patency after middle cerebral artery (MCA) recanalization [4]. The present study was conducted to evaluate the treatment effects of the diagnostic agent SonoVue® and a new microbubble agent, BR38, with regards to reversal of perfusion deficits after stroke caused by occlusions of the microcirculation. BR38 is especially designed for usage in therapy of acute intracranial artery occlusion. BR38 has been characterized in detail in Schneider *et al* [12]. Details of the respective properties and characteristics of SonoVue® and BR38 are outlined in Table 1.

**Table 1:** Properties of SonoVue® and BR38 Microbubbles,

	SonoVue®	BR38
Gas	100% sulfur hexafluoride (SF_6_)	35% perfluorobutane (C_4_F_10_), 65% nitrogen (N_2_)
Stabilizer	phospholipid	phospholipid
Charge	negative	neutral
Microbubble diameter	2.5 μm (0.7–10.0 μm)	1.4 μm (< 1.0–6.0 μm)
Resuspension with saline	5.0 ml	2.5 ml
Number of microbubbles in resuspension	4 x 10^8^	4 x 10^8^
Full dose	4 doses i.v., 15 min intervals	4 doses i.v., 15 min intervals
	10 μl of resuspension with 90 μl saline	10 μl of resuspension with 90 μl saline
1/3 dose	4 doses i.v., 15 minute intervals	4 doses i.v., 15 minute intervals
	33.3 μl of full dose with 66.7 μl saline	33.3 μl of full dose with 66.7 μl saline
Volume per application	0.1 ml	0.1 ml
Total volume administered	0.4 ml	0.4 ml

## Methods

### Animal Preparation

All procedures were performed in accordance with institutional guidelines and the German animal protection legislation and were approved by the regional animal care and use committee (Regierungspraesidium Darmstadt; Az. B2/257). Rats were administered 5 mg/kg Carprofen (Rimadyl™, Pfizer, Germany) subcutaneously 30 minutes prior to surgery, anesthetized with 5% isoflurane and maintained during surgery via a facial mask with 2–3% isoflurane in air (0.5 L/min). Rectal temperature was maintained at 37.0°C (±0.25°C) with a circulating water heating pad.

**Fig 1:**
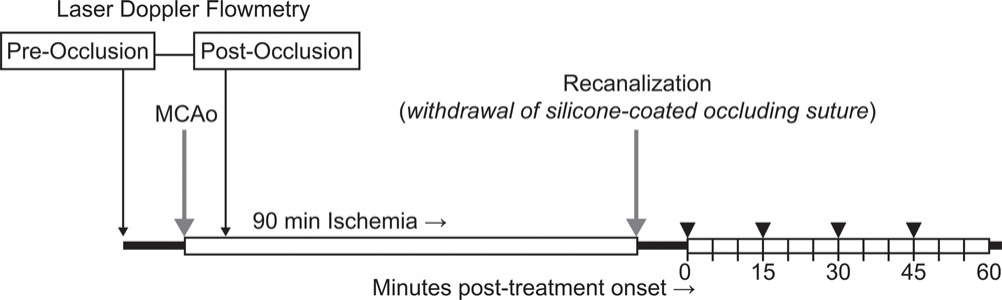
Experimental timeline. Microbubble enhanced sonothrombolysis was tested in a model of 90 minute MCA occlusion. Regional cerebral blood flow (rCBF) was measured by laser doppler flowmetry to confirm successful occlusion (post-occlusion). Randomization to treatment group occurred post-recanalization and immediately pre-treatment onset. rt-PA was administered every 5 minutes (dashed lines) in all treatment animals. Microbubbles (SonoVue or BR38) were administered at 15 minute intervals (▼). Control and rt-PA treatment groups received equal volumes of saline for rt-PA and microbubbles. Continuous ultrasound was applied for 60 minutes in conjunction with rt-PA and microbubble infusion.

A total of 44 male Wistar Unilever rats (Harlan Winkelmann, Germany) weighing 275–350 g underwent 90-minute right hemispheric MCA occlusion as previously described [4]. Briefly, the right common carotid artery was exposed and a silicone-coated nylon suture (4–0) was inserted and advanced proximally until mild resistance was felt. This indicated that the suture tip had reached the anterior cerebral artery, occluding blood flow to the right MCA (18–22 mm beyond the carotid bifurcation). At 90 minutes post-occlusion, recanalization of the MCA was established by withdrawing the suture. Success of occlusion was confirmed by Laser Doppler Flowmetry (LDF) (OxyFlo 2000®; Oxford Optronix, England) of the regional cerebral blood flow (rCBF). For LDF, the skull was exposed after local anesthesia with 2% Lidocaine (Xylocain®, AstraZeneca, Germany). rCBF was measured as described by Soehle *et al* [13] at 15 locations above the MCA territory measured pre-occlusion (baseline) and post-thread insertion to confirm occlusion (Fig 1). LDF values were recorded as blood perfusion units (BPU) prior to suture insertion and occlusion was confirmed by a drop in BPU to <1000.

### Experimental Groups

Immediately following withdrawal of the occluding filament at 90 minutes, animals were randomized to six treatment groups (n = 6/group): control, rt-PA, or rt-PA+3-MHz ultrasound insonation with BR38 or SonoVue® at full or 1/3 dose (Table 1). An ultrasound or tPA+ultrasound group were not included because these were previously compared to microbubble treatment [4]. Randomization was done in a blinded manner so the surgeon was unaware of treatment allocation. Treatment duration was 60 minutes.

Full dose SonoVue® was calculated by comparing the microbubble concentration in the blood volume of humans with the concentration in the blood volume of rats. The bubbles per ml of full dose BR38 equates to the bubbles per ml of full dose SonoVue® (Table 1). Treatment agents were resuspended in saline and administered via the tail vein. To permit dosing of microbubbles through the same catheter, 10 mg/kg rt-PA (Actilyse®; Boehringer Ingelheim, Germany) was delivered by small bolus injection every 5 minutes over 1 hour. The first injection was equivalent to a 10% loading dose. SonoVue® and BR38 (Bracco Suisse SA, Switzerland) were administered at four time points (Fig 1). Animals in control and rt-PA groups received equal volumes of saline.

A 3-MHz diagnostic ultrasound probe was placed 40 mm above the skull (B-mode, color-Doppler functions switched on, maximum output (mechanical index of 1.7)) (Sonos 7500; Philips Ultrasound, USA). The distance between the skull and ultrasound probe was bridged with ultrasound gel (Sonosid®; Asid Bonz, Germany). The beam was aligned to expose the entire brain (spectral Doppler sample volume placed in the midbrain (57 mm)). In the control and rt-PA groups, the probe was positioned but not turned on.

### Post-mortem Preparation

Animals were deeply anesthetized with inhaled isoflurane (5%) post-treatment and transcardially perfused with heparinized saline. Ligations were placed at the origin of the aortic arch, between the left common carotid and subclavian artery, on the innominate artery and the left external carotid artery. A radiopaque agent (Microfil®; Flow Tech, USA) was infused through the aortic arch for the *in situ* preparation of the cerebral vessels, the brains were harvested and fixed in formalin until analysis.

### Micro- and Nano- Computed Tomography (CT)

The X-ray system has previously been described in detail [14] (SkyScan® 1072_80kV; Belgium). The method of analysis was as previously described [4] with a few modifications. Briefly, a coronal aligned section of 3 mm thickness was cut out of each brain for detailed scans of the region with an isotropic voxel size (11.6 μm side length), encompassing the MCA territory (S1 Fig). The total contrast agent volume represents the sum of all pixels marked as contrast agent after thresholding. The total contrast agent volume equals the total vascular volume (mm³). Regions of interest (ROI) were manually determined in the right and left hemispheres to encompass striatum and cortex using Analyze™ 9.0 (Biomedical Imaging Resource, Mayo Clinic, USA). These regions were chosen as they are the most commonly affected regions in this stroke model. ROI templates were applied to each scan that were equally sized for cortex or striatum and positioned equidistant from the midline in respective regions and hemispheres. The vascular volume fraction (VVF) of each hemisphere was the sum of the cortical and striatal ROIs for that hemisphere, and represented a fraction of the total vascular volume. Nano-CT scans were performed on 2 samples (control and BR38 full dose) to illustrate changes in small arteries in the cortical region at high resolution. A cylindrical sample with a diameter of 2 mm was scanned using nano-CT (Skyscan 2011, Bruker Micro-CT, Kontich, Belgium) with a spatial resolution of 2.5 μm isotropic voxel side length. The nano-CT system has previously been described in detail [14, 15, 16].

### Histology

After micro-CT analysis, brains were trimmed using a matrix, embedded in paraffin, sectioned at 5 μm and stained with hematoxylin and eosin. Microscopic examination was performed by a veterinary pathologist experienced in brain tissue histology in a blinded fashion. As typical infarcts are not expected so early in the development of the lesion, the acute ischemic changes (AIC) were characterized by vacuolation of the nervous tissue and/or degenerating neurons (S2 Fig). Areas with AIC were measured using NIS Elements software (version 1.10, Nikon). AIC volumes were calculated from the section encompassing the MCA territory.

### Inclusion and exclusion criteria

Animals were included if post-occlusion regional cerebral blood flow (rCBF) declined to <1000 BPU. Prespecified exclusion criteria were: unstoppable bleedings during surgery (n = 1), perforation of the skull bone during laser Doppler flowmetry (LDF) preparation, inability to advance the occluding filament more than 18 mm beyond the carotid bifurcation, subarachnoid hemorrhage (SAH) caused by the occlusion (n = 2), obliteration of the tail vein during treatment delivery (n = 2), or insufficient Microfil® perfusion as judged by perfusion of the contralateral hemisphere (n = 3). These criteria were not associated with treatment effect and each excluded animal was replaced.

### Statistical Analysis

Statistical analysis was performed using Microsoft Excel 2010 (Microsoft Corporation, Redmond, Washington, US) and Graphpad Prism 6 (Graphpad Software, Inc. CA, USA). Micro-CT of VVF in hemispheres were analyzed using two-sided, paired t-test. LDF post-occlusion is presented as a percentage of pre-occlusion baseline. Differences between groups were analyzed by one-way ANOVA with Bonferroni post-hoc analysis for multiple comparisons. Acute ischemic changes were analyzed using Kruskal-Wallis with Dunn’s multiple comparison test. Data are presented as mean±s.d. and *p*<0.05 was considered significant.

## Results

Occlusion of the MCA by filament insertion resulted in an rCBF drop to 59.2±13.0% of baseline for all animals (n = 36). One-way ANOVA with multiple comparisons comparing all means and comparing all groups to control showed no significant differences between groups (p>0.05). Individual group means were: control 55.0±6.6%, rt-PA 55.1±13.9%, BR38 full dose 71.3±3.3%, BR38 1/3 dose 64.2±10.7%, Sonovue® full dose 51.5±16.1%, and Sonovue® 1/3 dose 58.1±15.7%.

### Micro- and Nano CT

**Fig 2:**
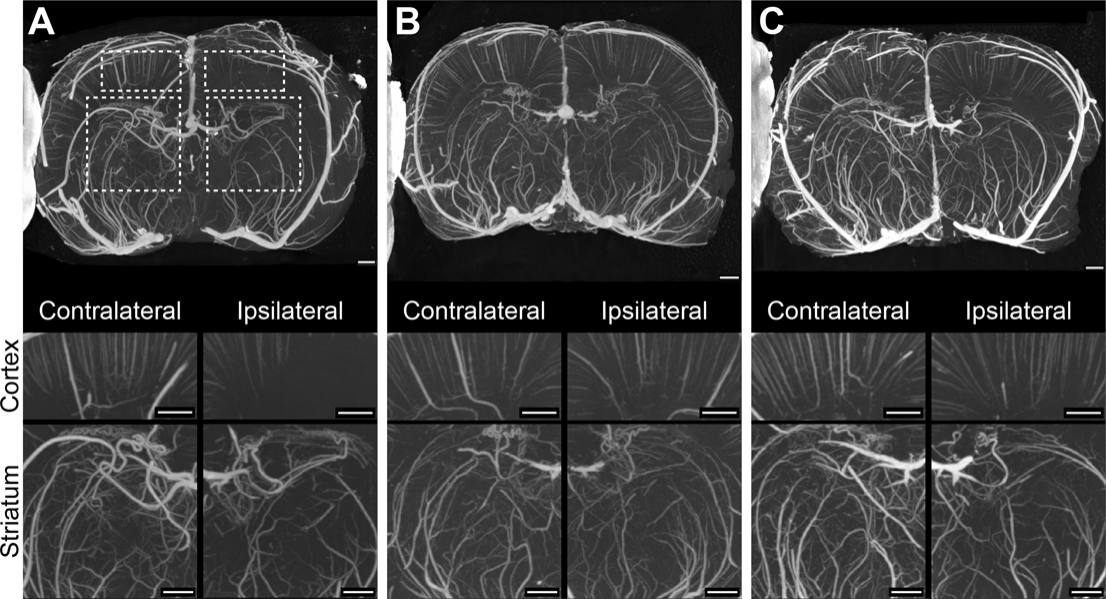
Micro-CT scan for vascular volume. Representative scans of the 3 mm slice encompassing the MCA territory used for micro-CT analysis from a control (saline treated) (panel A), a BR38 full dose (panel B) and a SonoVue full dose treated rat (panel C). Cortical and striatal regions of interest (ROI) for all animals were analyzed in each hemisphere to quantify vascular volume. Dashed line boxes in panel A have been drawn to demonstrate the ROI chosen for this animal. These scans demonstrate reduced vascular volume in both cortical and striatal regions of the ipsilateral hemisphere of the control rat, while demonstrating normal microvascular perfusion after BR38 or SonoVue microbubble-enhanced sonothrombolysis. Scale bar in each image = 800 μm.

**Fig 3:**
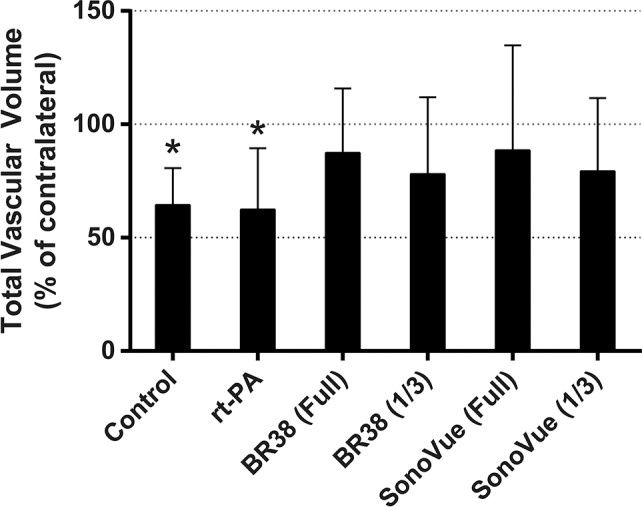
Vascular volume of the ipsilateral hemisphere. Total vascular volumes (VV) of the ipsilateral hemisphere quantified from micro-CT scans are presented as a percentage of the vascular volume of the contralateral (left) hemisphere. Control and rt-PA ipsilateral total VV were significantly reduced from contralateral total VV. * *p*<0.05.

**Fig 4:**
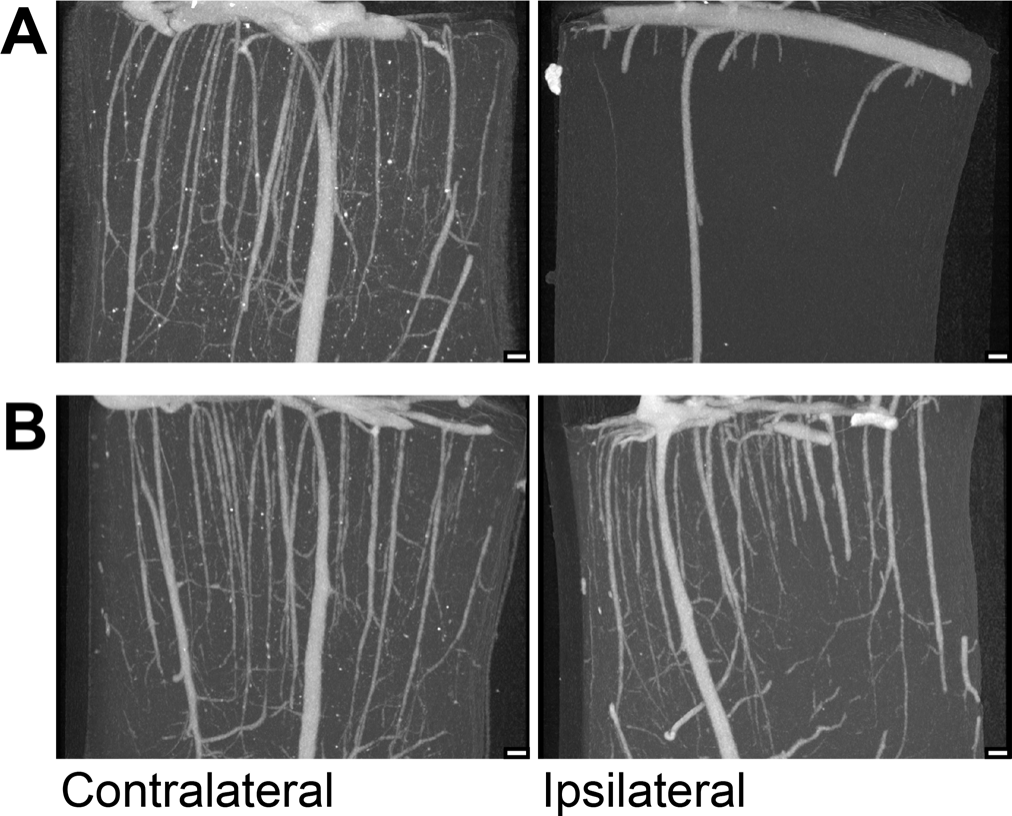
Nano-CT scan of cortex. Nano-CT images illustrating the “no-reflow phenomenon” in an untreated animal after the withdrawal of the MCA filament (A), and reperfusion of the microvasculature after BR38 (full dose) sonothrombolysis. Scale bar in each image = 100 μm

The VVF of control and rt-PA treated groups was significantly reduced in the infarcted hemisphere compared to the corresponding contralateral hemisphere (58% and 60% respectively, p<0.05) (Fig 2). The VVF of the ipsilateral hemisphere of all microbubble-sonothrombolysis groups was not significantly different from the contralateral hemisphere (Fig 3). Fig 4 is an illustrative nano-CT scan showing higher resolution of the cortical region of a control and BR38 full dose animal to demonstrate greater microvessel patency in the microbubble-sonothrombolysis treated animal.

### Histology

**Fig 5:**
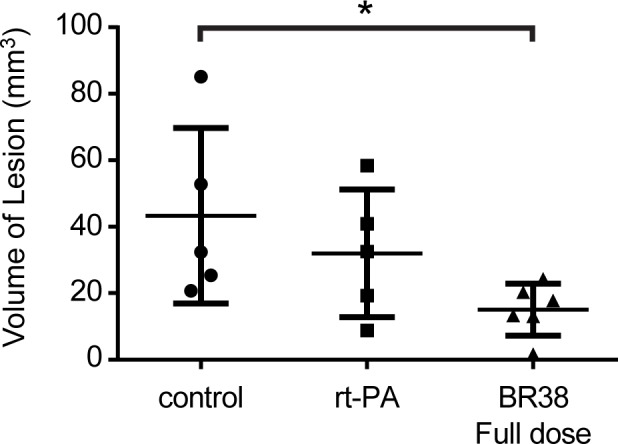
Volume of ischemic lesion. The volume of acute ischemic changes was measured from 3 mm thick brain sections encompassing the MCA territory. Data indicate a reduction of lesion volume following treatment with BR38 full dose combined with rt-PA and ultrasound. *p = 0.044.

Histological examination was performed on control, rt-PA and BR38 full dose groups. One animal was excluded from analysis due to too many post-mortem artifacts (rt-PA group). AIC were observed in all animals and were mostly detectable within the 3 mm brain section encompassing the MCA territory (striatum and/or cortex). The AIC volume was 15.1 ± 7.9 mm^3^, 32.0 ± 19.2 mm^3^ and 43.3 ± 26.4 mm^3^ for the BR38 full dose, rt-PA and control group respectively (Fig 5) with a significant difference between control and BR38 full dose groups (*p* = 0.044). The presence of the radiopaque agent, Microfil, within the blood vessels made examination for hemorrhage difficult, however hemorrhage was observed in 3 animals. Slight focal hemorrhage around a thrombosed vessel was observed in two animals: rt-PA (n = 1) and BR38 full dose (n = 1) groups. Another animal (rt-PA group) exhibited slight to moderate multifocal SAH as well as minimal to slight multifocal perivascular hemorrhage within the ischemic tissue. Reanalysis of VVF and AIC excluding the animal with SAH made no difference to significance.

## Discussion

We have demonstrated improved microvascular patency in a stroke model of cerebral “no reflow” following microbubble-enhanced sonothrombolysis. We found no difference in the effectiveness of two microbubble formulations, BR38 and SonoVue®, irrespective of dosage. Our data demonstrate that deficits of microvascular patency can persist after recanalization of major cerebral arteries and that rt-PA alone is not sufficient to improve this deficit. Currently, there are no specific therapies for microvascular occlusion, yet incomplete reperfusion occurs in approximately a quarter of patients with successful recanalization [9, 17]. Clinical reports now indicate that reperfusion, rather than recanalization, is a better predictor of good clinical outcome after acute ischemic stroke [9–11]. Hence, development of therapies that target both large- and micro-vessel occlusions may prove beneficial for improving patient outcome. Microbubble-enhanced sonothrombolysis could be a potential candidate.

Sonothrombolysis with BR38 microbubbles has been demonstrated to lyse large thrombi *in vitro* [18], but its effect on restoring microvasculature patency was unknown. Consistent with our previous micro-CT analysis of SonoVue®-enhanced sonothrombolysis [4], this study indicates that microbubble enhanced-sonothrombolysis may be beneficial beyond targeting the main thrombus, by also improving microvascular patency. This could be of particular benefit to patients whose main artery recanalizes but in whom hypoperfusion of the ischemic territory persists. This model was chosen because microthrombosis is known to occur after recanalization [4]. Reductions of rCBF reported in this study are not as large as conventional MCAo studies, however the interpretation of the rCBF drop with our method is different to conventional single point LDF. The averaging of rCBF over 15 locations over the MCA territory contributes to the relative drop appearing less than using a single recording site. Our method can take into account between-animal differences of the MCA distribution that single point LDF does not. Although rCBF drops were not a large as often reported in MCAo models, small vessel occlusion was appropriately induced with this model, as demonstrated by the reduced vascular patency of the ipsilateral hemispheres in the control and rt-PA groups. There was no significant difference in the rCBF drops in all groups and we therefore concluded that the improved microvascular patency of the microbubble-treated groups was a treatment effect. However, the onset time of treatment in our study limits translation of these results. Clinically, sonothrombolysis after recanalization, when recanalization is achieved with tPA, is not feasible given the increased haemorrhage risk. But the results of this study provide evidence that microbubble-enhanced sonothrombolysis restores microvascular patency and should prompt further investigation in to the potential clinical application of this strategy.

It was expected that BR38 microbubbles would improve microvascular patency to a greater degree because BR38 circulate for longer and are smaller in size than SonoVue®, therefore potentially reaching smaller vessels of the brain. Yet, microvascular patency was improved to the same extent, regardless of microbubble formulation or dosage. Clinical testing of various microbubble formulations (Levovist®, Definity®, SonoVue®) has demonstrated increased large vessel recanalization and improved functional outcome [1–3, 19]. Our findings correlate with two previous clinical studies comparing microbubble formulations [20] and dosages [1] that demonstrated comparable outcomes of recanalization rates and time to recanalization between treatment groups. This, together with our results, suggests a class effect of microbubbles for enhancing sonothrombolysis in both small and large vessels. Specific microbubble design or dosage may be less important. However, we did not see complete return to contralateral values of vascular volume, suggesting that despite microbubble therapy, there is still a small degree of persistent microvascular occlusion. It is unknown whether different ultrasound parameters, or longer duration therapy may return these vascular volumes to contralateral values completely, but the results of this study indicate that further investigation is warranted.

In this study we observed occlusion of the penetrating arterioles (Figs 2 and 4). Low perfusion pressures within penetrating arterioles due to capillary occlusion could cause incomplete perfusion of the contrast agent, overestimating total occlusion. Yet, a clear ipsilateral deficit is observed indicating that occlusions are related to the stroke. Our results suggest microbubble-enhanced sonothrombolysis can restore vascular filling but the exact location of thrombosis would require further investigation. Occlusion of arterioles has been implicated as a possible cause of lacunar infarction and evidence suggests occlusion of single penetrating arterioles results in significant neuronal death [21]. While it was not our intention to study lacunar infarction, with more preclinical testing, microbubble-enhanced sonothrombolysis may be a potential therapeutic for this condition.

Histological assessment of acute ischemic changes demonstrated reduced lesion size following microbubble-enhanced sonothrombolysis and restoration of vascular volume. This correlates with clinical evidence that although large vessel recanalization is associated with good outcome, reperfusion may be a more accurate predictor of final infarct volume [9–11]. Although histological results are in agreement with Micro-CT results, some limits do apply. The presence of Microfil and post-mortem tissue artifacts made the histological analysis challenging and in the section encompassing the MCA territory, some areas could not be evaluated for ischemic lesion due to the nature of the sectioning process for micro- and nano-CT analysis. Microfil perfusion was required for analysis of our primary outcome, forsaking vascular histology that might identify exact sites of thrombosis within arterioles and capillaries. Additionally, nano-CT provides higher resolution images and better definition of the microvessels, as illustrated in Fig 4, however because of the superiority of micro-CT for imaging the entire MCA territory without further manual sectioning of the tissue, it was chosen for quantification. Differences between microbubble formulations or dosages may have been more apparent on nano-CT had it been suitable for quantifying the whole MCA territory, since larger capacitance vessels may disproportionately influence total vascular volumes.

This study was designed to determine differences in efficacy of BR38 and SonoVue® at both equivalent and lower doses to the SonoVue® tested by Nedelmann et al [4]. For this study, we compared these formulations and doses against tPA-therapy to determine any benefit over current standard treatment. The decision not to include an ultrasound + rt-PA group without microbubbles was made because Nedelmann et al [4] demonstrated that although ultrasound + rt-PA improved ipsilateral patency, a perfusion deficit still persisted compared to the contralateral hemisphere. Contrast-enhanced ultrasound with SonoVue® + rt-PA was shown to restore ipsilateral patency to contralateral levels. Additionally, this study and the study of Nedelmann et al [4] are the first studies to investigate this therapy for reperfusion of microvascular occlusions. Hence, it was considered important to understand whether or not the mechanism worked before proceeding to the much larger trials that would be needed to examine functional outcomes. We purposefully investigated an early time point given that the more rapid the restoration of perfusion, the more likely the beneficial outcome to patients. Hence, sacrifice was early and without recovery of the animal from anaesthetic. Extended time windows to incorporate neurobehavioral function could confound the interpretation of efficacy data. The decision to limit the groups for histological analysis was also made during the study design due to the early sacrifice time. Typical ischemic changes were not expected to develop so early in the ischemic process and this early sacrifice is a likely cause of the large variability observed in Fig 5. Additionally, there was a possibility of artificial misinterpretation due to the microfil within the vessels.

We saw no difference in degree of hemorrhage in the BR38 microbubble group compared with rt-PA only, correlating with clinical stroke studies of high-frequency ultrasound microbubble-enhanced sonothrombolysis that report no significant increases of symptomatic intracerebral hemorrhage rates [22]. It appears that the concerns regarding excessive rates of bleeding first raised in the TRUMBI clinical trial [23], largely pertain to the use of low-frequency ultrasound [23, 24] and high-dose microbubbles [1]. This study is limited for assessing hemorrhage, specifically due to small numbers and the animal model. Caution should also be observed when comparing rat to human data for sonothrombolysis safety. The causes of haemorrhage related to ultrasound are likely to differ with respect to skull size and insonation field size. Because this was a study of reperfusion efficacy, correlation of these findings with safety outcomes should be performed in larger animals. Additionally, TCCS and higher dose microbubbles have caused increased hemorrhage rates for clinical large vessel sonothrombolysis [1, 25]. While the results of this study should prompt further study of sonothrombolysis for microvascular occlusion, safety outcomes should be assessed before clinical testing.

Our study indicates that microbubble-enhanced sonothrombolysis may restore perfusion deficits that persist even after large vessel recanalization in a model of acute ischemic stroke by restoring microvessel patency. This was observed in all microbubble-treated animals irrespective of microbubble formulation or dosage. This finding indicates the potential of this treatment strategy for future clinical use in patients with persistent occlusion of the microvasculature, for which there is currently no direct therapy.

## Supporting Information

S1 Fig Micro-computed tomography. Whole brain micro-CT (A) and resultant micro-CT image of section encompassing the middle cerebral artery territory (B). The red dashed box in (A) is representative of the section made from the whole brain tissue. Yellow arrows indicate the middle cerebral artery.
(PDF)

S2 Fig Acute ischemic changes. Representative example of the acute histological changes observed in the ischemic areas (panel B) characterized by vacuolation of the nervous tissue and degenerating/dead neurons. Panel C: non ischemic tissue. The dark material in capillaries is the radiopaque agent, Microfil (wide arrow, panel B).
(PDF)

S1 Text Minimal dataset of rCBF, vascular volume fractions and acute ischemic changes. (PDF)
