# Supplementary material for: Sonothrombolysis with BR38 Microbubbles Improves Microvascular Patency in a Rat Model of Stroke
Source: PLoS One. 2016 Apr 14;11(4):e0152898. doi: 10.1371/journal.pone.0152898 (PMC4831751; doi:10.1371/journal.pone.0152898)
Supplement: S1 Fig — Whole brain micro-CT (A) and resultant micro-CT image of section encompassing the middle cerebral artery territory (B). The red dashed box in (A) is representative of the section made from the whole brain tissue. Yellow arrows indicate the middle cerebral artery. (PDF) [file pone.0152898.s001.pdf]

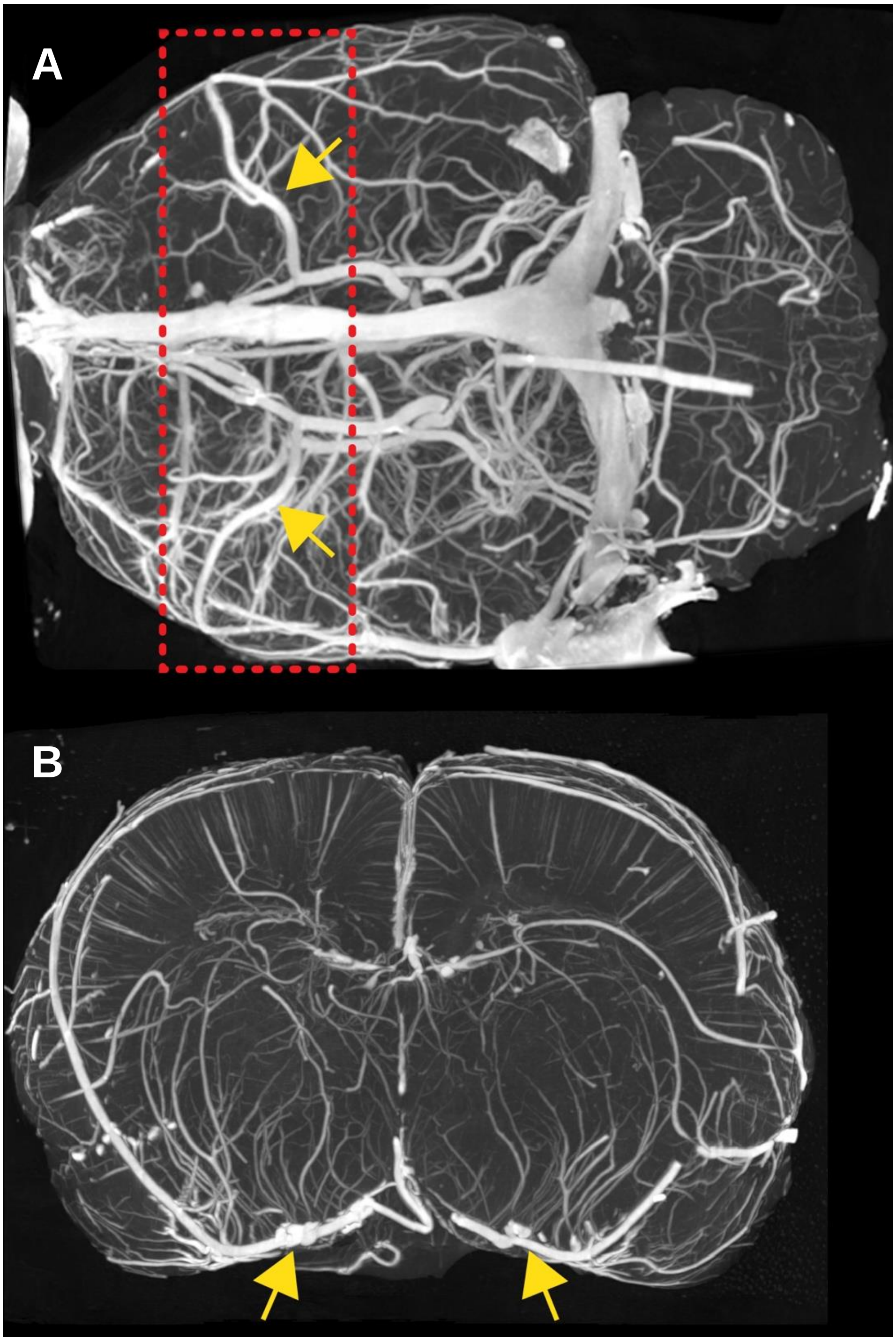

S1 Fig. Micro-computed tomography. Whole brain micro-CT (A) and resultant micro-CT image of section encompassing the middle cerebral artery territory (B). The red dashed box in (A) is representative of the section made from the whole brain tissue. Yellow arrows indicate the middle cerebral artery.
