# Supplementary material for: Sonothrombolysis with BR38 Microbubbles Improves Microvascular Patency in a Rat Model of Stroke
Source: PLoS One. 2016 Apr 14;11(4):e0152898. doi: 10.1371/journal.pone.0152898 (PMC4831751; doi:10.1371/journal.pone.0152898)
Supplement: S2 Fig — Representative example of the acute histological changes observed in the ischemic areas (panel B) characterized by vacuolation of the nervous tissue and degenerating/dead neurons. Panel C: non ischemic tissue. The dark material in capillaries is the radiopaque agent, Microfil (wide arrow, panel B). (PDF) [file pone.0152898.s002.pdf]

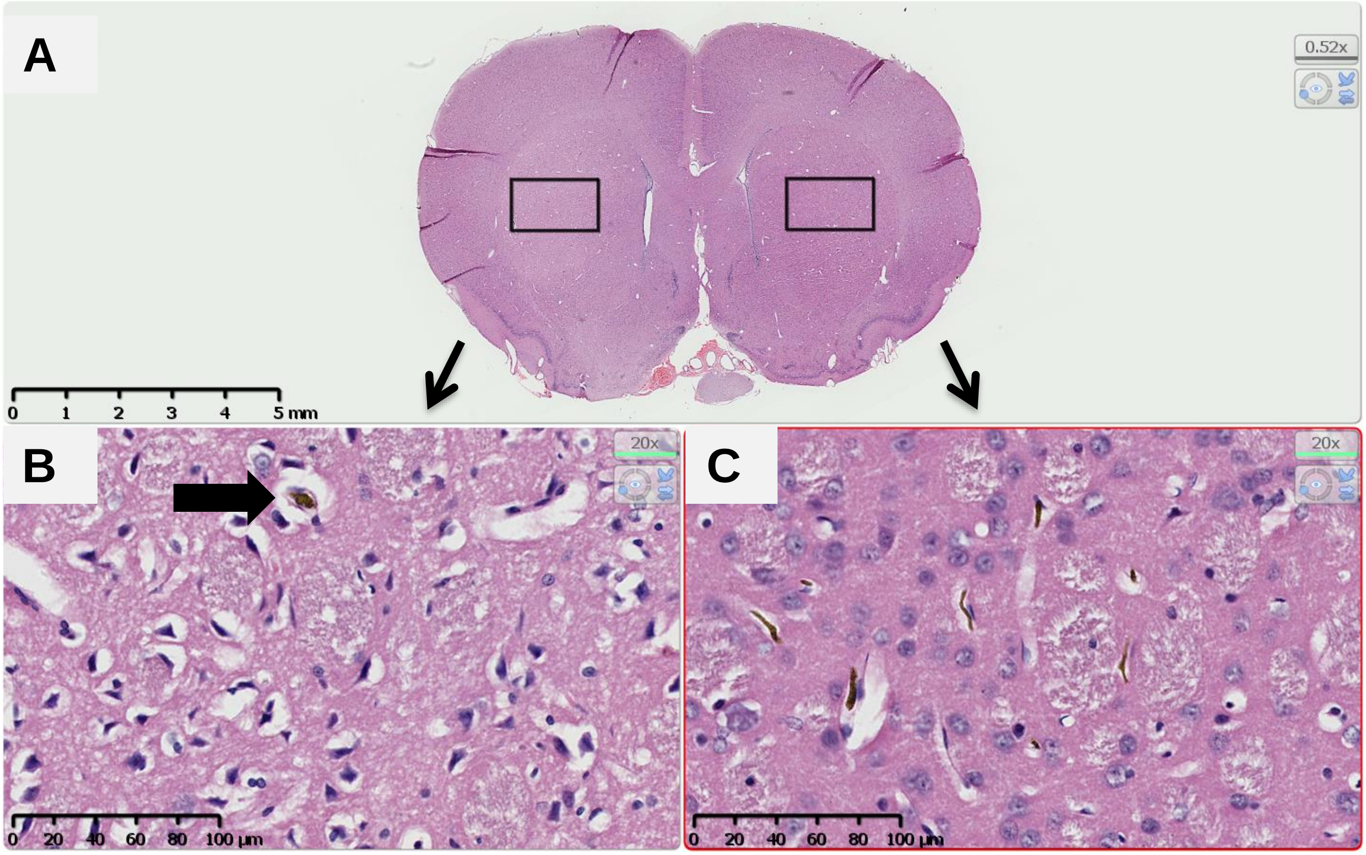


**S2 Fig. Acute ischemic changes.** Representative example of the acute histological changes observed in the ischemic areas (panel B) characterized by vacuolation of the nervous tissue and degenerating/dead neurons. Panel C: non ischemic tissue. The dark material in capillaries is the radiopaque agent, Microfil (wide arrow, panel B) .
