# Supplementary material for: Sonothrombolysis with BR38 Microbubbles Improves Microvascular Patency in a Rat Model of Stroke
Source: PLoS One. 2016 Apr 14;11(4):e0152898. doi: 10.1371/journal.pone.0152898 (PMC4831751; doi:10.1371/journal.pone.0152898)
Supplement: S1 Text — (PDF) [file pone.0152898.s003.pdf]

***Schleicher et al.*** *Sonothrombolysis with BR38 microbubbles improves microvascular patency in a rat model of stroke*

**S1 Text.** Minimal dataset of rCBF, vascular volume fractions and acute ischemic changes.

## Laser Doppler Flowmetry (LDF)

| Animal | Blood Perfusion Units (BPU) | | Post-occlusion flow as % of baseline | | |
|---|---|---|---|---|---|
| | Baseline | Post-occlusion | | Mean | SD |
| **Control (Saline treatment)** | | | | | |
| **4** | 1080 | 708 | 65.6% | **55.0%** | **6.6%** |
| **10** | 1266 | 670 | 52.9% | | |
| **28** | 1162 | 524 | 45.1% | | |
| **29** | 1254 | 699 | 55.7% | | |
| **33** | 1312 | 732 | 55.8% | | |
| **51** | 1141 | 625 | 54.8% | | |
| **rt-PA** | | | | | |
| **12** | 1202 | 838 | 69.7% | **55.1%** | **13.9%** |
| **16** | 1287 | 760 | 59.1% | | |
| **19** | 1081 | 729 | 67.4% | | |
| **31** | 1156 | 529 | 45.8% | | |
| **32** | 1241 | 692 | 55.8% | | |
| **35** | 1361 | 447 | 32.8% | | |
| **BR38 (Full dose)** | | | | | |
| **7** | 1114 | 807 | 72.4% | **71.3%** | **3.3%** |
| **9** | 1171 | 808 | 69.0% | | |
| **22** | 1029 | 778 | 75.6% | | |
| **27** | 975 | 719 | 73.7% | | |
| **46** | 1121 | 744 | 66.4% | | |
| **53** | 1064 | 751 | 70.6% | | |
| **BR38 (1/3 dose)** | | | | | |
| **13** | 1211 | 1061 | 87.6% | **65.6%** | **13.3%** |
| **26** | 1200 | 689 | 57.4% | | |
| **40** | 1223 | 620 | 50.7% | | |
| **42** | 1101 | 657 | 59.7% | | |
| **47** | 1155 | 740 | 64.1% | | |
| **49** | 1033 | 764 | 74.0% | | |
| **Sonovue (Full dose)** | | | | | |
| **14** | 1112 | 843 | 75.8% | **51.5%** | **16.1%** |
| **23** | 1148 | 672 | 58.5% | | |
| **43** | 1206 | 375 | 31.1% | | |
| **48** | 1048 | 393 | 37.5% | | |
| **50** | 1186 | 573 | 48.3% | | |
| **52** | 1203 | 692 | 57.5% | | |
| **SonoVue (1/3 dose)** | | | | | |
| **15** | 1207 | 964 | 79.9% | **58.1%** | **15.7%** |
| **20** | 1172 | 769 | 65.6% | | |
| **24** | 1122 | 421 | 37.5% | | |
| **36** | 1058 | 713 | 67.4% | | |
| **39** | 1309 | 594 | 45.4% | | |
| **45** | 1245 | 656 | 52.7% | | |

**S1 Text.** Minimal dataset of rCBF, vascular volume fractions and acute ischemic changes.

## Micro-CT Vascular Volume Fraction (VVF)

| Animal | Striatum | | Cortex | | Total Volume (mm3) (*Left hemisphere + Right Hemisphere*) | Right hemisphere as % Left hemisphere |
|---|---|---|---|---|---|---|
| | Left | Right | Left | Right | | |
| **Control (Saline treated)** | | | | | | |
| **4** | 0.4151 | 0.3559 | 0.0403 | 0.0169 | 0.8281 | 81.85% |
| **10** | 1.7217 | 0.8272 | 0.5598 | 0.0699 | 3.1786 | 39.32% |
| **28** | 1.1786 | 0.8309 | 0.3726 | 0.2129 | 2.5950 | 67.29% |
| **29** | 1.4955 | 0.7373 | 0.0779 | 0.0516 | 2.3622 | 50.14% |
| **33** | 1.3684 | 1.0260 | 0.2722 | 0.2090 | 2.8756 | 75.28% |
| **51** | 0.2174 | 0.2034 | 0.1103 | 0.0347 | 0.5658 | 72.65% |
| **Mean** | **1.0661** | **0.6634** | **0.2388** | **0.0992** | **2.0675** | **58.44%** |
| **St Dev** | **0.6103** | **0.3156** | **0.2019** | **0.0884** | **1.0995** | **49.00%** |
| **rt-PA** | | | | | | |
| **12** | 1.2007 | 1.0792 | 0.2848 | 0.2276 | 2.7922 | 87.96% |
| **16** | 1.1951 | 0.5186 | 0.3444 | 0.1250 | 2.1831 | 41.81% |
| **19** | 1.1346 | 0.6175 | 0.2319 | 0.2238 | 2.2079 | 61.57% |
| **31** | 1.6336 | 0.3787 | 0.3237 | 0.0710 | 2.4070 | 22.98% |
| **32** | 1.0955 | 1.0001 | 0.2872 | 0.2951 | 2.6780 | 93.67% |
| **35** | 1.7338 | 1.2804 | 0.4914 | 0.2037 | 3.7092 | 66.70% |
| **Mean** | **1.3322** | **0.8124** | **0.3273** | **0.1910** | **2.6629** | **60.47%** |
| **St Dev** | **0.2768** | **0.3572** | **0.0891** | **0.0802** | **0.5683** | **19.00%** |
| **BR38 (Full dose)** | | | | | | |
| **7** | 0.9079 | 0.7525 | 0.2332 | 0.1490 | 2.0425 | 79.00% |
| **9** | 0.8227 | 0.6904 | 0.2718 | 0.2204 | 2.0053 | 83.22% |
| **22** | 0.5375 | 0.9058 | 0.1972 | 0.1144 | 1.7549 | 138.86% |
| **27** | 1.2461 | 1.1889 | 0.2762 | 0.2956 | 3.0067 | 97.52% |
| **46** | 1.2632 | 0.8446 | 0.3309 | 0.1728 | 2.6115 | 63.83% |
| **53** | 0.7944 | 0.5879 | 0.2349 | 0.0558 | 1.6730 | 62.54% |
| **Mean** | **0.9286** | **0.8283** | **0.2574** | **0.1680** | **2.1823** | **84.01%** |
| **St Dev** | **0.2812** | **0.2092** | **0.0462** | **0.0835** | **0.5209** | **14.00%** |
| **BR38 (1/3 dose)** | | | | | | |
| **13** | 0.8240 | 1.0285 | 0.1665 | 0.2875 | 2.3065 | 132.87% |
| **26** | 1.3182 | 0.8802 | 0.2583 | 0.1665 | 2.6232 | 66.40% |
| **40** | 1.2823 | 0.7527 | 0.2958 | 0.0621 | 2.3929 | 51.63% |
| **42** | 1.0489 | 1.1542 | 0.2227 | 0.1863 | 2.6121 | 105.42% |
| **47** | 1.3410 | 0.8070 | 0.3384 | 0.2882 | 2.7746 | 65.21% |
| **49** | 1.0232 | 0.5077 | 0.1363 | 0.0397 | 1.7069 | 47.22% |
| **Mean** | **1.1396** | **0.8551** | **0.2363** | **0.1717** | **2.4027** | **74.62%** |
| **St Dev** | **0.2070** | **0.2252** | **0.0768** | **0.1064** | **0.3806** | **10.00%** |

**S1 Text.** Minimal dataset of rCBF, vascular volume fractions and acute ischemic changes.

| Animal | Striatum | | Cortex | | Total Volume (mm3) (*Left hemisphere + Right Hemisphere*) | Right hemisphere as % Left hemisphere |
|---|---|---|---|---|---|---|
| | Left | Right | Left | Right | | |
| **Sonovue (Full dose)** | | | | | | |
| **14** | 1.1212 | 0.7264 | 0.2027 | 0.2495 | 2.2998 | 73.71% |
| **23** | 0.6238 | 1.0246 | 0.1969 | 0.3612 | 2.2065 | 168.86% |
| **43** | 1.4970 | 0.7730 | 0.3768 | 0.1378 | 2.7846 | 48.61% |
| **48** | 0.7465 | 0.3339 | 0.1134 | 0.0254 | 1.2192 | 41.79% |
| **50** | 0.4348 | 0.4065 | 0.0497 | 0.0646 | 0.9555 | 97.23% |
| **52** | 0.4203 | 0.4144 | 0.0856 | 0.0965 | 1.0168 | 100.97% |
| **Mean** | **0.8073** | **0.6131** | **0.1708** | **0.1558** | **1.7471** | **78.62%** |
| **St Dev** | **0.4243** | **0.2712** | **0.1178** | **0.1266** | **0.7787** | **27.00%** |
| **Sonovue (1/3 dose)** | | | | | | |
| **15** | 0.8504 | 0.7932 | 0.0868 | 0.0725 | 1.8029 | 92.38% |
| **20** | 0.7403 | 0.4200 | 0.0191 | 0.0262 | 1.2056 | 58.76% |
| **24** | 1.0312 | 0.8835 | 0.2772 | 0.1075 | 2.2994 | 75.75% |
| **36** | 1.4356 | 0.6461 | 0.0259 | 0.0795 | 2.1871 | 49.65% |
| **39** | 1.0249 | 1.3212 | 0.2535 | 0.4381 | 3.0377 | 137.62% |
| **45** | 0.9715 | 0.7556 | 0.2561 | 0.0066 | 1.9897 | 62.09% |
| **Mean** | **1.0090** | **0.8033** | **0.1531** | **0.1217** | **2.0871** | **79.60%** |
| **St Dev** | **0.2373** | **0.2996** | **0.1221** | **0.1593** | **0.6043** | **71.00%** |
